# Tackling the epidemic of obesity and hypertension in children and young people

**DOI:** 10.1097/MNH.0000000000001156

**Published:** 2026-01-09

**Authors:** Emily Haseler, Manish D. Sinha

**Affiliations:** a Department of Paediatric Nephrology, Evelina London Children's Hospital, Guys & St Thomas’ NHS Foundation Trust; b Department of Clinical Pharmacology, School of Cardiovascular and Metabolic Medicine, King's College London, London, UK

**Keywords:** blood pressure, child, health policy, obesity, population

## Abstract

**Purpose of review:**

The prevalence of both obesity and hypertension is increasing in childhood, with considerable overlap in disease and causative factors. Evidence from studies over the past decade suggests both obesity and hypertension have summative effects on both cardiac remodelling in childhood and major adverse cardiovascular events in adulthood.

**Recent findings:**

We highlight recent high-quality evidence reporting epidemiology of obesity, hypertension and hypertension-mediated organ damage (HMOD) in those with obesity-related hypertension. We discuss the early life influences on BP and BMI trajectory and the clustering of cardiovascular risk factors (CVRFs) seen in obesity hypertension. We discuss management options highlighting key contributors to compounding risk for obesity-associated hypertension across the first two decades of life, with potential windows for both individual and population-level intervention.

**Summary:**

Currently, a large and expanding cohort of young people with multiple CVRFs is progressing toward adulthood, where they are likely to experience disproportionate cardiovascular morbidity when compared with age-matched healthy peers. These trends underscore the urgent need for co-ordinated healthcare responses to manage affected children and for robust governmental and public-health interventions to mitigate the environmental and societal drivers of this convergent epidemic.

## INTRODUCTION

Childhood obesity continues to rise globally. A recent meta-analysis including ~45.8 million children and adolescents from 2033 studies across 195 countries reported worldwide prevalences of 8.5% [95% confidence interval (CI) 8.2–8.8] for obesity and 14.8% (95% CI 14.5–15.1) for overweight [1^▪^]. Prevalence of obesity increased from 7.1% in studies published 2000–2011 to 11.3% in studies published 2012–2023, and higher prevalence was associated with higher national income and development indices [1^▪^]. Similar patterns are mirrored in childhood hypertension: global prevalence doubled between 2000 and 2020, increasing from 3.40% (95% CI 2.14–5.34) to 6.53% (4.17–10.07) in boys and 3.02% (1.90–4.75) to 5.82% (3.71–9.01) in girls [2^▪^].

Hypertension is significantly more common in children with excess weight: 16.35 and 6.79 vs. 2.57% for obese, overweight and normal weight, respectively. Obese children have systolic blood pressure (SBP) and diastolic blood pressure (DBP) approximately 7.5 (SD 3.4 to 11.6)/4.1 (2.1–6.1) mmHg higher than in normal-weight children [3,4^▪^]. Longitudinal Canadian data show through adolescence, each 1 unit BMI increase correlates with an increase of 0.7 mmHg in SBP [5]. While high-income countries (HICs) currently have the highest prevalence of both obesity and hypertension, low-income and middle-income countries (LIMCs) are experiencing the fastest increases, reflected in increased cardiovascular morbidity in young adults [6^▪^]. There is also global heterogeneity regarding prevalence of hypertension within obese cohorts and vice versa, likely due to a combination of environmental and heritable factors common to specific populations.

Diagnostic thresholds also influence epidemiology. The 2017 American Academy of Pediatrics (AAP) Hypertension guidelines introduced a single diagnostic threshold of 130/80 mmHg for adolescents 13 years and over, aligning with the US adult hypertension definition [7]. In addition, normative BP distributions for younger children were recalculated to exclude all overweight individuals, thus lowering diagnostic percentiles [8]. The 2016 European Society of Hypertension (ESH) guidelines for children and adolescents aged less than 16 years remain unchanged at at least 95th centile of either SBP or DBP compared to the normal population for age, sex and height and above 140/90 mmHg for those ≥16 years [9]. 

**Box 1:**
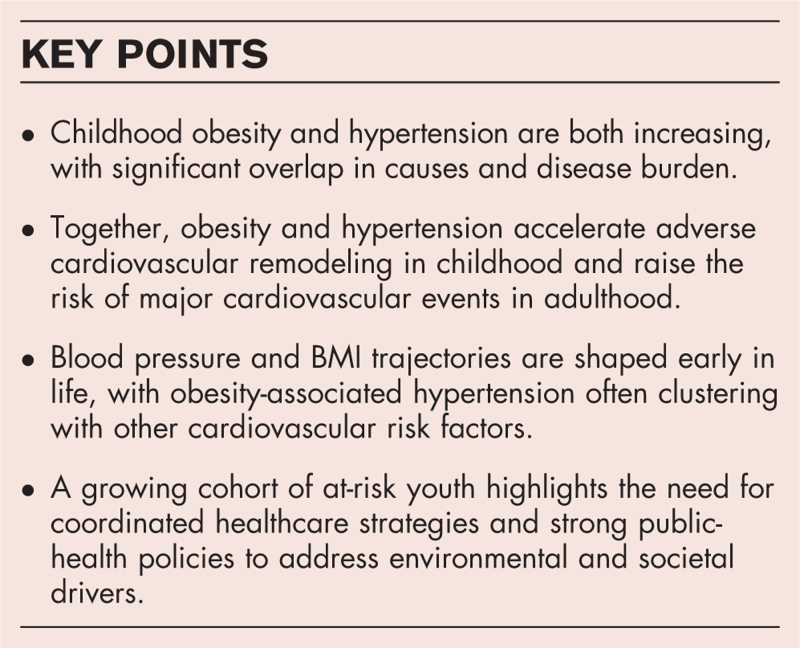
no caption available

## OBESITY, HYPERTENSION AND HYPERTENSIVE MEDIATED ORGAN DAMAGE

As major adverse cardiovascular events (MACE) are exceedingly uncommon in youth, studies have focussed on surrogate indicators of disease progression and early cardiovascular risk, particularly hypertensive mediated organ damage HMOD.

### Cardiac remodelling

Obesity and hypertension are each independently associated with raised indexed left ventricular mass (LVMI) and other markers of cardiac remodelling [10,11]. Over 20 years follow-up in young adults, a 1 kg/m^2^ increase in BMI carried a similar odds ratio for cardiac remodelling as a 10 mmHg increase in SBP [12]. A recent meta-analysis reported a 30.5% (95% CI 27.2–33.9) prevalence of left ventricular hypertrophy (LVH) in children with primary hypertension. Although the authors excluded studies with predominantly obese individuals, BMI was the only independent predictor of LVH [13]. Obesity and hypertension appear to promote differing patterns of left ventricular remodelling. Elevated BMI is associated with an eccentric pattern of remodelling (increased LV diameter and elevated LVMI with normal relative wall thickness) whereas hypertension is associated with a concentric pattern of remodelling (preserved LV diameter and LVMI, with elevated relative wall thickness) [14,15]. In combination, effects are additive, and concentric LVH is more prevalent in obese hypertensive than obese normotensive youth [16,17].

### Vascular remodelling

The most measured markers of vascular HMOD are pulse wave velocity (PWV) and carotid intimal–media thickness (cIMT), representing functional and structural vascular injury, respectively. Both have been shown in adult populations to be independently associated with MACE, and both are elevated in hypertensive children [18–20,21]. Population studies show complex interactions between adiposity, BP and vascular measures. The KiGGS (German Health Interview and Examination Survey for Children and Adolescents) study found a stepwise rise in PWV across normotensive, prehypertensive and hypertensive groups, and across normal-weight, overweight and obese groups, with additive effects when these risks co-occurred [22]. In contrast, ALSPAC (Avon Longitudinal Study of Parents and Children) data from the UK suggested lower PWV in obese 10-year-olds compared with normal-weight peers, but higher PWV in obese adolescents aged 17 [23,24]. Within a smaller ALSPAC subset, sustained high fat mass over 7 years predicted higher PWV at 17 years compared with consistently low or declining fat mass [23,24]. Lurbe *et al.*[25] found PWV decreased with increasing weight category in 8–18 year olds, whereas Stabouli *et al.*[26] reported PWV rose only when obesity and hypertension coexisted, with BMI alone not predictive. A systematic review including 12 studies found higher cIMT in hypertensive vs. normotensive children, although mean values (reported in one-third of the included studies) were in the normal range [21]. After further examination of multifactorial determinants, the investigators concluded determinants of PWV were predominantly BP-related, while cIMT was influenced by BMI, adiposity and metabolic–immune factors [21].

These findings suggest several possibilities: obesity and BP may influence PWV before structural vascular change occurs, vascular remodelling may begin early in childhood, or BP-independent mechanisms may also raise PWV.

### Microalbuminuria

Although obesity and hypertension increase chronic kidney disease risk in adults, microalbuminuria is inconsistently observed in children [22]. Interestingly, population studies show a positive association with BP but an inverse relationship with BMI [23].

## EARLY LIFE INFLUENCES ON BLOOD PRESURE AND BMI TRAJECTORY

Prenatal, intrauterine and neonatal exposures shape long-term BP and adiposity via ‘foetal programming’. Adverse developmental environments during critical windows alter metabolic and neuroendocrine pathways [24]. These are presumed to interact with genetics, environmental factors and childhood weight trajectory to determine long-term BP ‘set point’, propensity to gain weight and ultimately cardiovascular risk [25]. Early-life risk factors common to both obesity and hypertension include high prepregnancy maternal BMI, high maternal gestational weight gain, infants born small-for-gestational age (SGA), prematurity and rapid weight gain in the first 5 -years [26,27]. In particular, SGA and prematurity compounded by rapid early weight gain (termed ‘catch up growth’) are thought to confer the greatest risk on long-term cardiovascular health [28].

Interestingly, animal studies demonstrate central nervous system-mediated pathways, including hypertension response sensitisation (HTRS), where prior exposure to low-dose pressors primes an exaggerated hypertensive response. High-fat feeding induces HTRS in both mothers and offspring, and central renin–angiotensin–aldosterone system (RAAS) blockade prevents this, underscoring neuroplastic mechanisms linking obesity and BP regulation [29,30].

## TRACKING AND CLUSTERING OF CARDIOVASCULAR RISK FACTORS

Both BMI and BP show strong longitudinal tracking; children with high BMI and BP centiles are more likely to become obese and hypertensive adults [31–34]. BP tracking strengthens with age and is amplified by obesity, whereas weight trajectory in early childhood is the strongest determinant of later obesity, with most obese adolescents having become overweight before age 5 [32,33].

In the past few years, robust data has started to emerge regarding the impact of clustering of cardiovascular risk factors (CVRFs) on MACE in adulthood. Much of this is from the International Childhood Cardiovascular Cohort (i3C) consortium, an amalgamation of longitudinal population cohorts from Finland, USA and Australia spanning 20–40 years of follow-up in over 38,000 individuals [35]. Childhood CVRFs are cumulative, with each unit increase of a composite risk *z* score (encompassing SBP, BMI, youth smoking, total cholesterol and triglyceride level) associated with a hazard ratio of 2.71 (95% CI, 2.23 to 3.29) for adult MACE [35]. In 20,656 individuals in whom a fatat cardiovascular event could be ascertained, obesity and hypertension each independently predicted adult MACE with hazard ratios of 3.34 (2.42–4.60) and 2.04 (1.24–3.35), respectively[35]. Childhood BMI is suggested as the strongest determinant of adult cardiovascular risk and is nearly as important as adult BMI, whereas SBP risk is conferred via indirect pathways [36^▪^].

These data suggest the well recognized polygenic and environmental risk factors for hypertension and obesity are compounded by a variety of early life factors. Maternal BP and weight shape foetal growth; early-life growth patterns influence lifelong BP trajectories; and obesity makes BP trajectories more resistant to modification. Thus, it follows that critical windows for prevention should be as early as possible in the life course: during pregnancy for high-risk mothers, and in the preschool years for high-risk offspring. For example, increasing the physical activity of obese women during pregnancy found improved markers of cardiac remodelling in offspring at age 3 years [37].

## PATHOPHYSIOLOGY OF OBESITY-RELATED HYPERTENSION

Obesity promotes hypertension through interacting neurometabolic, immune and hormonal pathways. Visceral, adiposity promotes a chronic inflammatory state that drives insulin resistance and hyperinsulinaemia, all of which augment sympathetic nervous system (SNS) activity and BP [38,39]. Adipose tissue also produces angiotensin II and aldosterone, providing a direct link between obesity and RAAS activation [40]. Lastly, obesity contributes to endothelial dysfunction via altered nitric oxide and endothelin pathways, though some paediatric studies suggest heightened endothelial reactivity early in life [41,42].

Haemodynamic changes such as higher heart rate or cardiac output are frequently reported in youth with obesity, likely secondary to metabolic dysregulation and immune signalling and leading to SNS overactivity [39,41,43–45]. These plus dietary sodium excesses are likely to contribute to a volume and SNS driven ‘cardiac’ hypertension, increasing BP before substantial vascular structural change occurs.

## BLOOD PRESSURE MEASUREMENT CONSIDERATIONS IN OBESITY

Accurate BP measurement is challenging in obese children. Incorrect cuff sizing can misclassify BP, and auscultation is technically more difficult [38,46^▪^]. Obesity also reduces imaging quality in echocardiography and may limit feasibility of cardiac and vascular evaluation using MRI. Emerging cuffless blood pressure technologies may help address these barriers, but rigorous validation across the full range of paediatric ages and BMI categories is essential before they can be integrated into clinical care [47^▪^].

## TREATMENT

**FIGURE 1:**
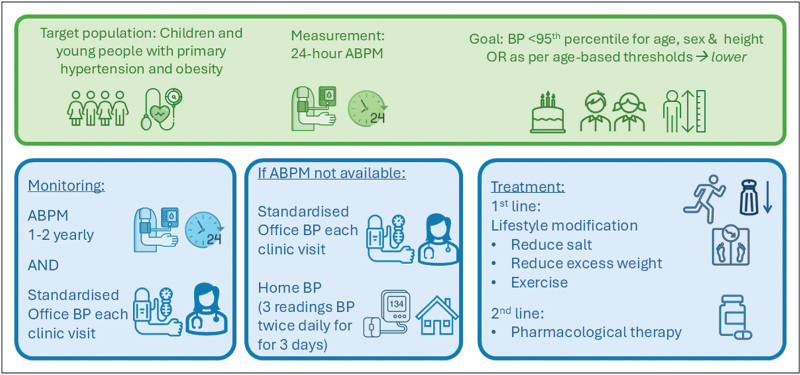
Initial measurement, monitoring and treatment principles in young people with hypertension and obesity. Adapted from Flynn *et al.*[7] and Lurbe *et al.*[9]. ABPM, ambulatory blood pressure monitoring; BP, blood pressure. ^*^Authors suggest targeting BP below <90th percentile.

**FIGURE 2:**
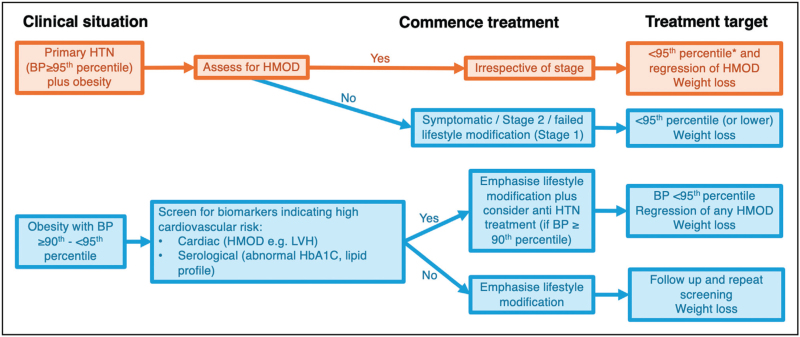
Suggested management pathway for young people with obesity and concerns regarding high blood pressure. Adapted from Flynn *et al.*[7] and Lurbe *et al.*[9]. BP, blood pressure; HbA1C, haemoglobin A1C; HMOD, hypertension mediated organ damage; HTN, hypertension; LVH, left ventricular hypertrophy. ^*^Authors suggest targeting BP below <90^th^ percentile.

The key clinical aspects of initial measurement, monitoring and treatment principles in young people with hypertension and obesity are outlined in Fig. 1 and summarized from international guidelines [7,9]. Nonpharmacological treatments remain the cornerstone of management for primary hypertension and those with high-normal blood pressure and should be continued even when pharmacological therapies are commenced [7,9]. Pharmacological management with antihypertensive medications are not discussed further in this article but are indicated if there is failure of nonpharmacological interventions, in the presence of symptoms, if the level of hypertension significantly elevated or in the presence of HMOD. Fig. 2 outlines the pathway for obese youth with BP equal to or greater than the 90th percentile for age, sex and height or as per age-based thresholds.

### Weight reduction

This is the single most effective intervention for BP control, target organ damage and ultimately long-term cardiovascular risk in obesity-related hypertension [7,9]. A meta-analysis showed lifestyle interventions of varying descriptions and durations were associated with weight reduction of −1.25 kg/m^2^ (95% CI −2.18 to −0.32) compared to usual care [48]. Seven studies in this analysis including 554 obese children reported SBP reduction of −3.40 mmHg (95% CI −5.19 to −1.61) and DBP −1.78 mmHg (95% CI −2.88 to −0.67) [48]. In severely obese children with mean age 11.4 ± 3 years and hypertension prevalence 39%, a lifestyle modification programme reported that a reduction in BMI *z* score of 1 (equivalent to BMI drop of ~3 to 4 kg/m^2^ for age and starting BMI) was associated with a reduction in SBP of 6.24 percentiles (95% CI 1.32 to 11.17; *P* = 0.02) and in DBP of 4.99 percentiles (95% CI 0.66–9.31; *P* = 0.03) [49^▪^].

Reductions in abdominal adiposity and increases in lean mass predict LVH regression in hypertensive adolescents [50,51^▪^]. Improvement-wider cardiometabolic profile, including total cholesterol, triglyceride, fasting insulin and HOMA-IR has also been observed [48,52]. Lastly, weight loss has been associated with reduction in salt sensitivity in children [53].

### Increased physical activity

Exercise programmes alone are typically associated with a 1–2 mmHg BP reduction, with combined interventions encompassing diet modification producing larger effects [54,55]. However, for those with comorbid obesity and hypertension, the BP reductions can be much greater. In one randomized controlled trial (RCT) involving four 90-min sessions per week over 1 year, prevalence of hypertension reduced from 86 to 16% [56].

### Focussed nutritional advice

The Dietary Approaches to Stop Hypertension (DASH) diet is recommended though paediatric evidence is modest [7,9,57]. This is perhaps not surprising given that a previous report from National Health and Nutrition Examination Surveys (NHANES), observed compliance with DASH advice to be low across all ages [58]. Interestingly, a trial in adolescents with BP greater than 90th percentile reported a 2.7 mmHg SBP reduction at 6 months, though benefits waned by 18 months despite sustained dietary improvements [59].

Long-term follow-up data are scarce, but one interesting study evaluated a relatively low-cost, twice-yearly dietary intervention delivered over 20 years and showed modest but measurable reductions in both SBP and DBP [60].

### Dietary sodium reduction

Reducing salt intake by around 40% in children and adolescents is associated with a BP decrease of about 1–3 mmHg [61,62]. Long-term follow-up of a neonatal salt-restriction intervention suggests early-life sodium exposure may influence lifelong blood-pressure trajectories, with sustained reductions in SBP ~3.5 mmHg even 15 years later [63]. An interesting study showed lowering salt intake by 1 g/day was associated with a 27 g/day reduction in intake of sugar-sweetened beverages, linking sodium reduction to obesity prevention [64]. This important observation highlights the relevance of reducing both salt and sugar consumption when managing obesity-associated hypertension. Like the experience with obese adults, a meaningful sustained lifestyle change remains difficult to achieve particularly in obese adolescents [65].

### Other lifestyle aspects

Stress reduction, improved sleep quality and smoking free environments are all also recommended in major guidelines to improve blood pressure control; however, evidence of impact is limited [7,9].

### Pharmacotherapy

Glucagon-like peptide-1 (GLP-1) receptor agonists have transformed obesity management in adults, with outcomes typically exceeding intensive lifestyle programme results [66^▪^]. The combination of appetite reduction from GLP-1 agonists and comprehensive lifestyle modification likely represents an optimal approach [67^▪^].

GLP-1 receptor agonists are now approved for use in selected adolescents 12 years and over with obesity and/or type 2 diabetes mellitus, under specialist services [68^▪^]. Use of semaglutide for 1 year was associated with a mean body weight reduction of 17.4% (−21.1 to −13.7) in the intervention compared to placebo group [69]. Trials involving liraglutide thus far show a smaller effect, although liraglutide has also been shown to be effective and well tolerated in children of 6–12 years [70▪–72^▪^]. Small reductions in BP (1–3 mmHg) have been reported, though no studies specifically involving obese hypertensive children have been performed [69,73]. Adult data suggest potential cardiovascular benefit via weight-dependent and independent mechanisms such as natriuresis, reduced inflammation and improved endothelial function [74^▪^,75^▪^]. Though GLP-1 agonists appear to have an improved safety profile in adults compared to surgical treatment options, their long-term use has not yet been fully evaluated. Gastrointestinal side effects, high treatment costs and posttreatment rebound weight gain must be carefully assessed in the context of the huge healthcare burden of obesity-related morbidity. Several other novel nutrient-stimulated hormone-based therapies in the pipeline for obesity treatment and will likely have similar limitations [66^▪^]. These issues will need specific focus when considered in children and young people in the future.

### Public health strategies and population-level interventions

**FIGURE 3:**
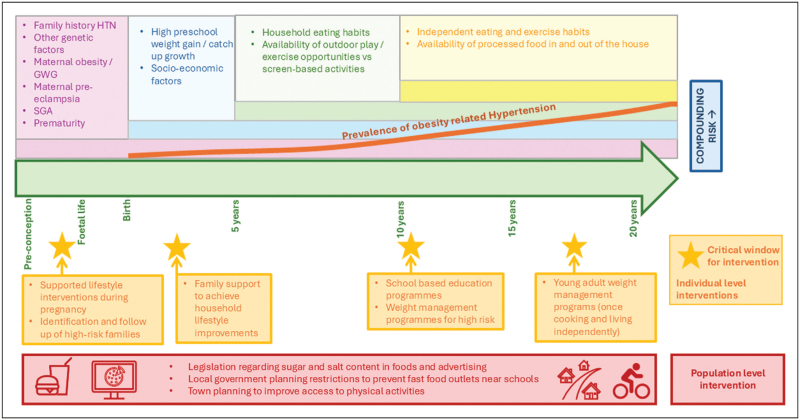
Key contributors to compounding risk for obesity-related hypertension across the first two decades of life, with potential windows for both individual-level and population-level intervention. GWG, gestational weight gain; HTN, hypertension; SGA, small for gestational age.

The use of public health strategies to reduce an ‘obesogenic environment’ for children and young people are central to prevention. Policies to reduce both salt and sugar exposure are likely to show most benefit in both LMICs and HICs [76,77]. Town planning to improve public transport, increase green spaces and promote outdoor exercise opportunities have also all been effective [78^▪▪^,^79^]. There is limited availability for health professionals even in HICs to provide sustained support for behavioural changes to be maintained and promote lifestyle interventions. These are evident with scarce high-quality multidisciplinary weight-management services for children and young people and overburdened clinical services on a background of chronic dis-investment in public health infrastructure. Fig. 3 summarizes key contributors to compounding risk for obesity-related hypertension across the first two decades of an individual's life course, with potential windows for both individual and population level intervention.

## CONCLUSION

There is no doubt that over the past decade, significant progress has been made in our understanding of the factors that influence obesity and hypertension and their clinical relevance from early life and over the life course. Despite substantial developments, several knowledge gaps remain with unanswered key questions. Future work to address these is needed to adequately tackle the epidemics of obesity and hypertension in children and young people. A combination of precision medicine with population health intervention approaches that utilizes data from digital health tools and learns from longitudinal cohorts from a young age are likely to provide most learning and inform future interventions.

## Acknowledgements

*None*.

### Financial support and sponsorship

*E.H. was funded by Medical Research Council Clinical Research Training Fellowship Grant number MR/X001768/1.*

### Conflicts of interest

*There are no conflicts of interest.*

## REFERENCES AND RECOMMENDED READING

Papers of particular interest, published within the annual period of review, have been highlighted as:

▪ of special interest
▪▪ of outstanding interest
